# The Behaviour Changes in Response to COVID-19 Pandemic within Malaysia

**DOI:** 10.21315/mjms2020.27.2.5

**Published:** 2020-04-30

**Authors:** Eugene Koh Boon Yau, Nicholas Pang Tze Ping, Wendy Diana Shoesmith, Sandi James, Noor Melissa Nor Hadi, Jiann Lin Loo

**Affiliations:** 1 Department of Psychiatry, Faculty of Medicine and Health Sciences, Universiti Putra Malaysia, Selangor, Malaysia; 2 Faculty of Medicine and Health Sciences, Universiti Malaysia Sabah, Sabah, Malaysia; 3 Department of Social Work and Social Policy, School of Science, Health and Engineering, La Trobe University, Australia; 4 Department of Psychiatry and Mental Health, Hospital Tuanku Fauziah, Perlis, Malaysia; 5 Queensway Clinic, Central and North West London NHS Foundation Trust, Milton Keynes, United Kingdom

**Keywords:** COVID-19, mental health, behaviourism, psychiatry, social sciences

## Abstract

The novel coronavirus infection, COVID-19, is a pandemic that currently affects the whole world. During this period, Malaysians displayed a variety of behaviour changes as a response to COVID-19, including panic buying, mass travelling during movement restriction and even absconding from treatment facilities. This article attempts to explore some of these behaviour changes from a behaviourist perspective in order to get a better understanding of the rationale behind the changes.

## Introduction

The novel coronavirus infection, otherwise known as COVID-19, has become the latest pandemic that has affected the whole world. After it was first described in December 2019 in China, Malaysia had their first confirmed case on the 25 January 2020 and the first confirmed death on the 17 March 2020. This was just one day before the country was placed under a strict movement restriction order (MRO). The number of cases has steadily increased since then. Two particular public events have been associated with a huge spike in confirmed cases (1).

The onset of the illness within Malaysia triggered some major changes in behaviour amongst the population; ranging from panic buying to the nonchalant approach of carrying on daily life despite the MRO. The reasons behind different behavioural changes are yet to be assessed in depth using systematic research methods. Hence, the authors attempt to analyse and provide some initial explanation for these behaviours from a behaviourist perspective.

## Behaviourism in Illness

Behaviourism views external physical actions and internal states like thoughts and emotions as a ‘behaviour’ (2). Relational frame theory states that humans learn and build experience by relating one idea over another to form a complex network that shapes how one thinks and makes choices (2). If a person were to be bitten by a dog, that individual might subsequently develop a fear of dogs or might end up as a very outspoken advocate for dogs. This psychological adaptation is essential to ensure survival as part of evolutionary psychology. The direction of where an individual ends up depends very much on how the individual associates and connects the dog bite into his or her internal network of thoughts. Their subsequent future choices when facing a similar situation will be influenced by the very same internal network of relations.

The authors would like to postulate the following network of association that explains the behaviour changes during COVID-19. Some ideas are universal for everyone. The idea that death is irreversible is one such universal thought. The idea that illnesses are bad is also universal. Linking both ideas, everyone has the association that they need to avoid illness to prevent death. This association is essential from an evolutionary perspective. The person that avoids illness avoids death and survives to pass on the same idea to their offspring. What an individual does with that idea within their network of relations, on the other hand, varies from individual to individual. An individual might take drastic steps to avoid the physical illness or to avoid being anxious about the illness. Another individual might work together with others to confront the illness despite the cost of death. This idea of illness and death plays a considerable role in shaping the behaviour and actions expressed by individuals during the COVID-19 period and will be referenced multiple times in this article.

## Behaviour Changes of the General Public

Initially, it has been observed, the general public typically responds to disaster with denial. It is a behaviour that can happen in an individual or group at times and is commonly discussed as a psychological strategy occurring in people who are facing difficult circumstances or events—in the face of unrelenting and unavoidable danger, or experiencing a perceived painful emotion. The use of denial, either as a deliberate choice or otherwise, functions as a method to avoid facing or dealing with the danger at hand; thus putting the danger out of awareness. It is an extensive use of cognition inhibition to avoid processing the overwhelming emotion and thoughts that accompany the issue at hand (3). One does not have to look far for examples of this phenomenon. In situations of sudden death, we are often confronted by the sight of a grief-stricken relative flatly denying that their loved one has passed on, with neither emotion nor expression. This behaviour can be extrapolated to those who flatly refuse to be tested for COVID-19 even though the evidence is overwhelming they have contracted it or are at high risk from contacts. There are also those who stopped others—family members, cohorts—from getting tested, as getting tested may result in unwanted outcomes that reinforce the denied fear of illness or potential death. Even during the period of MRO, multiple reports of non-compliance appear on a daily basis. This behaviour causes a likely increase in the spread of the illness as infected individuals continue with their daily life. This mechanism may result in significant disruption in health-seeking behaviour, which can be illustrated by the case of a family who attempted to escape quarantine by absconding from a hospital (4).

On the note of self-preservation, one behaviour that is quite inextricably linked to unusual psychological reactions in COVID-19— bulk-purchasing toilet paper (5). Dry or canned food supplies, instant noodles, mineral water or cooking oil are highly rational choices of things to hoard in a potential lockdown. Puzzlingly, though, many stores worldwide have run out of toilet paper as the significant hoarding item. Many explanations abound for this behaviour, all of which can only remain hypotheses at this juncture. Cognitively, in a crisis, ‘groupthink’ and ‘conformity’ tend to take precedence over individual acts of non-conformity that may prove to be lifesaving. People can panic when they observe others buying certain products, even if unrelated, as a mass fear infects individual to individual and no one wants to be left out of owning one non-essential item that appears to be running out. The panic buying is consistent with the model suggested by Mawson (6) where if the anxiety experienced is not at a severe level and attachment figures are absent, e.g. clear authority directions, the choice of behaviour usually move towards familiarity; which in this scenario, where ‘others’ think that bulk buying toilet paper is the appropriate behaviour. This choice was most likely driven also by media sources reporting the behaviour. Shifting focus onto an individual perspective, the authors postulated that the COVID-19 symptoms of coughing, digestive issues and runny nose are symptoms that commonly require the use of toilet paper. Individuals might associate toilet paper as a tool to prepare for or cope with the infection should they be unfortunate enough to be infected by it. Toilet paper is also an item that an individual can easily take up and store for a long time. The physical act of storing can act as a choice of behaviour that the individual can make to keep anxiety at bay. In the end, stocking up on toilet paper does provide relief to the individual’s fear and anxiety about COVID-19 symptoms.

Other human behaviours are more amenable to explanation. In a crisis, despite repeated warnings that people should not mass travel or congregate at transport terminals in a panic to buy travel tickets, in Malaysia we have witnessed people surging onto highways, onto buses and planes, trying to get themselves to their hometowns in the initial deregulated period (7), before full restrictions of movements were enforced by the police. This may be an act of familiarity; going home to one’s hometown is seen as a predetermined safe choice. It is also consistent with Mawson’s model that states that if the anxiety experience increases significantly, individuals are driven to seek out and get closer to safe attachment figures, e.g. family. This behaviour is seen time and time again in times of crisis and disasters (6).

Religion took a hot seat in Malaysia during the pandemic. While the COVID-19 pandemic did not drive attendance, one particular religious mass gathering during the COVID-19 period resulted in a large number of positive COVID-19 cases within Malaysia (8). There was also resistance seen among social media when there were suggestions in changing various regular religious practices. There is limited literature on the current pandemic. However, there are studies done during previous disasters. Religion is seen as a source of information and leadership. Amidst a time of uncertainty, individuals seek out direction by turning to religious figures (9–10). This again is consistent with Mawson’s model of anxiety and attachment figures (6). Religion also provides a source of explanation why things are happening, i.e. why is COVID-19 happening. The idea that everything that happened is part of a higher power’s plan is a form of reasoning and coping that an individual can use (10). Considering that religion is usually deeply rooted in the network of associations from a very young age in Malaysia and many other countries, choosing religion is often a natural choice. While the relationship between religion and scientific explanations can either be complementary or conflicting, one study (10) reported that individuals are still predominantly keen to take action to address the disaster they experienced. This is also reflected in COVID-19 where a majority of attendees at the previously mentioned gathering presented for testing (11) soon after the announcement of MRO.

## Discrimination During COVID-19

Discrimination from stigma, either public or self-stigma, during COVID-19, is another behaviour that can be seen (12). The formation of stigma is commonly conceptualised as a label that is associated with firmly held belief, usually false or exaggerated, and the resulting negative attitude and behaviour towards the label (13). COVID-19 is the label here that is associated with stigma. An individual does not need to have the infection to suffer from discrimination, just the mere suspicion of it suffices. Even healthcare providers experience discrimination of social stigma due to their exposure to the infection (14). Going back to the network of associations, the label of COVID-19 will inevitably be associated with the idea of death; and the discriminative reaction is the choice of behaviour to avoid being infected. However, this choice of behaviour often has detrimental effects on those suffering from discrimination. They could be socially ostracised, denied access to essential services, and receive physical and emotional abuse. They are also at risk of experiencing depressive and anxiety symptoms (14–15). The experience of elf-stigma can also result in reluctance in seeking help when necessary (16). The World Health Organization (WHO) had released a series of messages for various groups that addresses steps on minimising stigma; including judicious use of reference terms, limiting exposure to distressing news and finding opportunities to be supportive to others (17).

## Behaviour Changes of Healthcare Providers

There have been many forms of altruistic behaviour that have emerged in the COVID-19 crisis. Many doctors, members of the public and other frontline workers have volunteered to serve even though it is not their duty, or have volunteered to bring food and other necessary supplies to the frontline. At the same time, many members of the public have been proactive in posting ‘public service messages’ to encourage others to stay at home and comply with social distancing. The behaviour of altruism is a difficult concept to define, with many explanations being put forth. The choice of altruism could be a solution to avoid the progression of the unpleasant scenarios; to achieve interpersonal equilibrium; a deep underlying compassionate need to help or a responsibility that is expected from the society (18). For example, healthcare workers were also reported to be reluctant in seeking help. The behaviour of not seeking help was explained as a result of either a denial of being unwell, fear of being infected and uncertainty on how to act upon being infected (21). Anecdotal reports from one of our authors (Hadi) noted that healthcare workers with a possible risk of exposure are worried about getting tested themselves. There is the fear that testing positive will cause a possible backlash from surrounding colleagues exposed to them and the crippling of service as a result of massive quarantine. Regardless of theory, people who choose altruism as a reason to expose themselves to an illness were found to experience less psychological trauma subsequently (19). The choice of altruism also received many accolades from society (20).

Altogether, these behaviours reflect the underlying psychological state of people, mainly fear and anxiety. There are other mental health concerns to be considered such as depression, substance use disorder and post traumatic stress disorder following the aftermath of these emotional disturbances that may occur throughout and after the pandemic period. In response to this, steps must be taken to minimise the risk during and after the pandemic (22). Resilience has been found to correlate with the risk. Individuals with higher resilience were found to have reduced risk of developing psychopathology. It is suggested that psychological first aid or cognitive behaviour therapy-based intervention can help in reducing the psychological impact of the pandemic (22). Given the number of individuals affected and anticipated barriers, any psychological interventions delivered must be easy to use, self-instructive, adaptable to the situation, minimal in cost, of brief duration, and focused on techniques instead of theoretic principles. The authors, Shoesmith and James, had created such an intervention in 2018 named Brief Psychological Interventions for the Malaysian Setting (Unpublished), and the same intervention was adapted for use during COVID-19.

## Conclusion

The behaviour changes amongst Malaysian’s as a response to the COVID-19 pandemic are both novel and expected at the same time. Past literature on previous crises and disasters reported similar themes in behaviour changes, but the choice of behaviour changes are explicitly adapted to the COVID-19 pandemic. When viewed from a behaviourist perspective, the changes adopted are rational, even when they appear otherwise. They are influenced and shaped by the network of associations that an individual has or shared with others. They are also shaped by the goals of the individual, whether this is to avoid illness, avoid anxiety or to live by important life values. Some of the changes are useful, but because of excessive use, causes maladaptive consequences. By being mindful with the choices made and focusing on how useful or valuable the behaviour is, more adaptive behaviour can be made consciously. While the authors attempt to explore from the lenses of behaviourism, there are many other forms of psychological explanation that can be used to address the behaviour changes. These explanations are meant to provide a more substantial theoretical concept to an otherwise subjective topic in order to address the pandemic better. There is a need to explore these changes with further qualitative and quantitative studies for a better understanding of the behaviour changes specific to COVID-19 pandemic. These results can direct us to prepare better management for future similar pandemics.
